# Real Greatness

**Published:** 1874-10

**Authors:** 


### REAL GREATNESS,

On the part of individuals, communities, and states, is measured by the amount of human suffering abated and human enjoyment promoted in proportion to numbers. In the things which exhibit the human character in its grandest forms Boston takes the lead, and has kept it for a hundred years. Boston made the Revolution out of a rebellion; it was only success that prevented it from being the latter. Boston abolished slavery, giving equal rights to millions. Nothing short of a Boston could possibly have said, when half her millions were burnt in a night, we love the nation for its generosity, but we respectfully decline help to begin again. There is scarcely a human device for the amelioration of human suffering, and for the prevention of sorrow and sin, which has not had its initiative in Boston breasts, in building homes for infants, for decrepid age, for the unfortunate girl and for the homeless widow, for the poor old man without kith or kin, and the wife who had seen better days; for the drunkard, for the idiot, and in short for all who merit human help. In numberless directions has she shown the higher humanities in her efforts to prevent people from needing help; not to mention the old plans, scarce a year passes but some new method is devised and proposed for public consideration to help the less fortunate of their brothers and sisters to avoid poverty and earn an easy competence. Among the latest of these is the organization of societies called

THE SOVEREIGNS OF INDUSTRY, the two distinguishing features of which are to encourage the man who works for his money to avoid debt, and to show him how he may purchase all his supplies at the very lowest wholesale rate, and getting for his money the very best article of its kind, thus always shunning "cheap things," which, the world over, is at once the temptation and the loss of the poor.

The general plan is to form a society of fifty or a hundred, representing perhaps as many families, each of which is furnished with a token that he is a member. The president goes to the best wholesale establishments, and ascertains their lowest prices to the trade, and says, I want fifty barrels of flour, but I will not take all at once; and instead of sending them to me, and then I send them to my customer, he will call on you when he wants a barrel, you send it to his place, and he will pay you the money on delivery, I thus represent fifty families. But when a member wants a barrel of flour, he goes to the wholesaler; the retail price is, say eleven dollars; he orders it home; on its arrival he shows his card, which entitles him to two dollars deduction; and a plan is in preparation to go further back still, and find the man who makes the flour, and get him to send it to his commission merchant at a still further reduction of a dollar or two; thus the consumer saves the profits which would go to the retailer, and also that which goes to the commission merchant, for the most part, at least, causing a saving to the hard working mechanic of three or four dollars in a single barrel of flour, the wages of a day or two. In this way the members of the Order get their sewing machines at half price, and if the wife or daughter wants anything in the shape of a musical instrument, from a Jew's harp to a thousand dollar piano, she makes a saving of thirty-three and a third per cent.; and in dry goods, and every article which a family can eat, drink, or wear, there is a saving of from ten to thirty per cent., with the inestimable advantage, not only of getting the best things lower than the worst of their kind, but of keeping out of debt. And it is to be hoped that it will not be long before there will be a society of the "Sovereigns of Industry" in every county, if not in every village, in the whole land, resulting in lessening the toil of every hard working man, increasing his means, adding to the comfort and enjoyment of his family, and thus elevating them to a higher place in life. All honor to the wise heads and generous hearts of Boston who first devised this plan for the good of the hard working poor.

